# The Importance of Lassa Fever and Its Disease Management in West Africa

**DOI:** 10.3390/v16020266

**Published:** 2024-02-07

**Authors:** Rachel A. Reyna, Kirsten E. Littlefield, Nathan Shehu, Tomoko Makishima, Junki Maruyama, Slobodan Paessler

**Affiliations:** 1 Department of Pathology, University of Texas Medical Branch, Galveston, TX 77555, USA; 2 Department of Microbiology and Immunology, University of Texas Medical Branch, Galveston, TX 77555, USA; 3 Infectious Disease Unit, Department of Medicine, Jos University Teaching Hospital, Jos P.M.B. 2076, Nigeria; 4 Department of Otolaryngology, University of Texas Medical Branch, Galveston, TX 77555, USA

**Keywords:** arenavirus, Lassa virus, sensorineural hearing loss

## Abstract

Lassa virus (LASV) is a zoonotic pathogen endemic throughout western Africa and is responsible for a human disease known as Lassa fever (LF). Historically, LASV has been emphasized as one of the greatest public health threats in West Africa, with up to 300,000 cases and 5000 associated deaths per year. This, and the fact that the disease has been reported in travelers, has driven a rapid production of various vaccine candidates. Several of these vaccines are currently in clinical development, despite limitations in understanding the immune response to infection. Alarmingly, the host immune response has been implicated in the induction of sensorineural hearing loss in LF survivors, legitimately raising safety questions about any future vaccines as well as efficacy in preventing potential hearing loss. The objective of this article is to revisit the importance and prevalence of LF in West Africa, with focus on Nigeria, and discuss current therapeutic approaches and ongoing vaccine development. In addition, we aim to emphasize the need for more scientific studies relating to LF-associated hearing loss, and to promote critical discussion about potential risks and benefits of vaccinating the population in endemic regions of West Africa.

## 1. Introduction

Lassa virus (LASV) is the zoonotic pathogen responsible for causing Lassa fever (LF), a potentially hemorrhagic human disease endemic throughout western Africa [1]. This Old-World arenavirus predominately infects *Mastomys natalensis* as its major reservoir host, although recent work has identified LASV within *Hylomyscus pamfi* and *M. erythroleucus* rodents as well, with the virus shedding into their urine and feces [2,3,4,5,6]. The synanthropic nature of this rodent species, combined with its ability to shed the virus continuously, is highly conducive to LASV spillover into human populations [7]. Human infection is typically mediated through the inhalation or ingestion of infected rodent excreta and secreta or by direct contact with the bodily fluids and secretions of LF patients [4,8]. As a result, human-to-human transmission has been observed in nosocomial settings [4,9]. Currently, 37.7 million people across 14 countries in West Africa are at risk of infection [10]. However, the habitat of the *M. natalensis* rodent is continuously expanding throughout sub-Saharan Africa, which dramatically increases the risk of spreading LASV into populations previously not at risk that would be severely underprepared for such outbreaks [6]. This emphasizes the crucial need for surveillance and the development of safe and efficacious preventive measures and therapeutics.

According to the American Centers for Disease Control and Prevention (CDC), 100,000 to 300,000 LASV infections occur annually, resulting in 5000 deaths [4]. However, these case numbers are likely overestimated and do not represent the current status in West Africa. Nigeria represents a hot spot of LASV infection, yet even within this country the greatest number of annual confirmed cases was only 1189 in 2020 [11]. Moreover, a recent 2018 outbreak in Nigeria was considered unprecedented, with only 633 cases, albeit with a 27% case fatality rate [12]. Unfortunately, more accurate metrics on LASV incidence and burden remain challenging to obtain. Clinical diagnosis of LF is often confounded both by other pathogens that cause acute febrile illness as well as by the lack of accessibility to equipment for on-site diagnostics [13,14,15,16,17]. Additionally, LASV surveillance is highly inconsistent, occurring opportunistically and often capitalizing on pre-existing disease surveillance infrastructure, resulting in hyper-fixation on known endemic regions [18]. Therefore, the true number of LASV infections is currently unknown but may be lower than the previously published estimates.

LF disease progression is characterized first by nonspecific “flu-like” symptoms that devolve into neurologic and/or hemorrhagic manifestations in severe cases, which occur in approximately 20% of infections [13,19,20]. Though the overall case fatality rate of LF is estimated at around 1%, this number increases exponentially in hospitalized cases, ranging from 15 to 70% depending on the outbreak [4,21,22,23,24,25]. Individuals that survive LASV infection suffer a variety of life-long sequelae, the most pervasive of which is sensorineural hearing loss (SNHL), which occurs in approximately one-third of cases [26]. Despite its high incidence, the exact mechanism driving LASV-induced hearing loss remains unknown. However, recent work in an animal model of LF indicates it occurs in a CD4 T cell-dependent manner [27]. The high prevalence of sudden SNHL in survivors and potential involvement of the host immune response in its development raise serious concerns about vaccine development, the primary concern being that vaccine candidates invoking a robust immune response to LASV could induce SNHL in vaccine recipients. The greatest public health impact would result from efficacious, safe vaccines preventing both acute disease and hearing loss due to infection.

To date, no Food and Drug Administration (FDA)-approved vaccines or therapeutics exist for LF. However, off-label use of ribavirin has been shown to decrease the mortality associated with LF when administered within the first six days post onset of symptoms, although the high rate of misdiagnosis and late presentation make this timeframe difficult to meet [28]. In order to address perceptively high infection rates, national agencies have been pushing for the development of vaccines against LASV. Since 2015, the World Health Organization (WHO) has listed LASV amongst its priority pathogens requiring research and development efforts [29]. As a result, the Coalition for Epidemic Preparedness Innovations (CEPI) prioritized LASV for vaccine development funding [30]. Currently, CEPI is overseeing the generation of six LASV vaccine candidates in addition to supporting “The Enable Lassa Research Programme”, which seeks to gather epidemiological data on LASV in West Africa to prepare for vaccine clinical trials [30]. However, the urgency of rushed development of LASV vaccines is diminished in our opinion when considering the realistic case numbers, the success of ribavirin in treating LF, and, most importantly, the potential risks associated with any massive vaccination campaign in West Africa.

One of objectives of this article is to present LASV incidence in Nigeria by analyzing historical and more recent numbers. We also intend to emphasize the need for prolonged vaccine testing to ensure the safety of recipients and to exclude the possibility of SNHL in vaccine recipients. Moreover, we wish to encourage vaccine developers to include the prevention of LF-induced hearing loss as one of the primary or secondary endpoints in their clinical trial design.

## 2. Current Case Numbers

The American CDC estimates that each year, there are 100,000–300,000 new LASV infections and up to 5000 associated deaths in endemic regions [4]. However, these case numbers might be overestimated as they do not represent the current status in West Africa. These widely used numbers are extrapolations based on serological data published in 1987 in a study conducted exclusively in the eastern province of Sierra Leone [31]. Its extrapolations did not consider that 80% of Lassa infections are asymptomatic despite resulting in seroconversion, potentially causing an overestimation of symptomatic cases [14,15,17]. In addition, the authors themselves acknowledged that Sierra Leone may represent a LASV hot spot, causing their results to greatly overestimate the burden of LASV infections occurring in West Africa [31]. Subsequent research confirmed this concern, demonstrating that LASV has two endemic foci in West Africa, one in Sierra Leone and Liberia, and another in Nigeria [7].

Despite being a LASV hot spot, Nigeria alone has seen fewer confirmed cases in 52 years combined than the predicted yearly case number of 100,000–300,000. In this article, we will focus on LASV statistics reported from Nigeria where our team is actively working in hospitals with LF patients. Nigeria is used as an example for case numbers in this paper based on the size of the country, quality of the health system, and personal experience of the authors, who have worked on LF in Nigeria for many years. Between 1969 and 2021, there were only 25,191 suspected and 3897 confirmed cases of LF in Nigeria, resulting in 1319 deaths [32]. This averages out to fewer than 500 suspected cases, around 75 confirmed cases, and around 25 deaths per year. Between 1969 and 2017, the highest annual number of reported LF cases in Nigeria was still only 1022 cases during the 2016–2017 outbreak [33]. In 2020, this number increased to 1189 [11]. The year of 2018 featured an unprecedented outbreak in Nigeria, with 1893 suspected cases, 423 confirmed cases, and 106 fatalities between January and May [34]. Between April of 2018 and March of 2020, only 534 patients were admitted to Nigerian hospitals with confirmed LF [35]. Therefore, the real case numbers are likely substantially lower, as clearly demonstrated through publicly available hospital data from Nigeria.

Importantly, while case numbers may be dramatically lower than expected, case fatality rates may in fact be higher. The overall case fatality rate in Nigeria between 1969 and 2021 was 33.8%, much higher than the CDC-estimated 1% [32]. The highest reported Nigerian case fatality rate between 1969 and 2017 was 58.1% in a 2003 outbreak [33]. More recently, the January to May 2018 outbreak featured an overall case fatality rate of 25.1% [36,37,38]. The greatest case fatality rate published for LF was observed in Sierra Leone at Kenema Government Hospital, where 69% of antigenically positive patients (detected virus antigen in blood) succumbed to the disease between 2008 and 2012 [24]. These reports suggest that, though fewer infections may be occurring than was previously estimated, the infections that do occur are more likely to be lethal.

When ranking the public health importance of LF, it is important to also consider other diseases endemic to the area (Table 1). In Nigeria alone, around 100 million cases and 300,000 deaths are reported due to malaria each year [39]. Between 2011 and 2015, Nigeria featured a 27% positivity rate of malaria among its population [40]. While case numbers are difficult to obtain due to underreporting, dengue virus is also persistent in Nigeria, with between 10 and 67% of the population testing positive for neutralizing antibodies against the three subtypes of dengue virus in different regions [41,42,43]. Around 390 million dengue cases globally are estimated annually, with approximately 96 million being subclinical [43]. Between 2017 and 2019, yellow fever virus was responsible for 7894 suspected and 287 confirmed cases of yellow fever, another potentially hemorrhagic disease, across Nigeria [44]. Water-related diseases such as diarrheal disease, typhoid, and cholera also impose major burdens on the healthcare infrastructure, with diarrheal diseases alone being responsible for 16% of childhood fatalities in Nigeria each year [45,46]. The year of 2010 featured a severe outbreak of cholera throughout Nigeria, with around 3000 cases and 781 deaths reported [47]. The relative public health impact of these diseases endemic to Nigeria are summarized in Table 1. Apart from the obvious health implications, these diseases place a major socioeconomic burden on endemic countries due to surveillance and mitigation efforts. Between the higher case numbers of these diseases and their societal costs, LF does not pose the greatest threat to public health in western Africa.

While the elevated case fatality rate of LASV infection is evident, yearly case numbers are dramatically lower than those of other diseases endemic to the area. Continuing research efforts to understand the increase in case fatality rates of LF, the mechanisms driving pathogenesis and sequelae, as well as the development of vaccines and therapeutics are important. However, when comparing the threat posed by other endemic diseases to those benefitting from allocated funds, redistributing monetary support to address infections impacting a greater patient population may be justified.

## 3. Current Therapeutics

Off-label administration of ribavirin is currently the only available and widely used therapeutic for LF [48,49]. Ribavirin is a guanosine nucleoside analogue whose exact mechanism of action in inhibiting LASV infection remains unknown [50]. Previous work indicates that high levels of viremia are correlated with poor prognosis [13,17,28,51]. Ribavirin is markedly effective in reducing virus titer in patients and has been repeatedly shown to increase patient survival [23,50,52,53,54,55]. Treatment is recommended to commence as soon as a probable case is identified due to the primary effective window of ribavirin being constrained to the first six days post onset of symptoms [28,48]. Patients receiving ribavirin follow either the original McCormick regimen or the more modern Irrua regimen over a timeframe of 10–11 days; clinicians are at liberty to choose between either of the regimens as indicated by the Nigeria Centre for Disease Control [28,48,56]. There has been no direct clinical comparison of the efficacy of the two regimens. The main difference between the two regimens is dosage size; patients following the Irrua regimen receive a higher initial loading dose followed by lower doses administered once per day [48,56]. The McCormick regimen has a higher total ribavirin dose (417 mg/kg) administered to patients compared to the Irrua regimen (287 mg/kg) [57]. The Irrua regimen has the advantage of less frequent dosing, a lower total cost of ribavirin for the patient, lower risks of side effects, and less exposure to LF patients for healthcare workers. Both regimens exceed the NHP equivalent dose which was able to reduce mortality to 0% [57].

While the clinical research backing the use of ribavirin may be dated, the application of the drug has made a markedly positive impact in LASV-endemic areas. The pre-eminent study championing ribavirin as an effective treatment for LF was published in 1986 and demonstrated that intravenous administration of ribavirin within six days post onset of symptoms decreased mortality from 55% to 5% [28]. These results were supported by the cumulative findings of five retrospective clinical reports published between 2012 and 2018, which showed that treatment with parenteral ribavirin reduced LF’s mortality rate from 83% to 38.2% [23,50,52,53,54,55]. Additionally, preclinical studies involving nonhuman primates showed 100% protection against lethal challenge when administered prior to 7 days post infection [58,59,60]. However, these in vivo studies were conducted in the 1970s and 1980s by only one group of investigators. A recent study in cynomolgus macaques demonstrated an increased time to death due to ribavirin treatment, though protection against mortality was not observed [61]. The discrepancy in these findings may be attributed to several experimental factors, such as differences in viral isolates and animal breeding. Despite these promising results, several reports criticizing ribavirin’s use for the treatment of LF have been published, citing the need for unbiased preclinical and clinical studies to illustrate safety and efficacy [50,57,62]. Nevertheless, the ongoing discussion on ribavirin’s antiviral efficacy does not prevent clinicians from prescribing it to almost every LASV patient diagnosed in endemic areas.

Other broad-spectrum antivirals are being evaluated as potential treatments for LF. Favipiravir, an RNA-dependent RNA polymerase inhibitor shown to have antiviral properties against a wide array of viruses, displayed promising antiviral activity in animal models [63,64,65] and has been used in combination with ribavirin to successfully treat two clinical cases of LF [66]. Currently, favipiravir is in clinical trials to assess its safety and tolerability for the treatment of LF [67]. The viral entry inhibitor LHF-535 has also been shown to have potent anti-LASV efficacy in animal models [68]. Phase I clinical trials have already been completed demonstrating the safety and pharmacokinetics of LHF-535 in healthy human subjects [69], though phase II trials have yet to be initiated. As these antiviral alternatives continue to be developed, further research is required to assess their anti-LASV effectiveness in comparison to and in combination with ribavirin.

In Nigeria, the cost of healthcare is primarily out of pocket for the patient. Although the government recognizes the burden of LASV infection and helps subsidize bills, on average up to 61.8% of the cost, the high cost of treatment leads to patients often waiting until they are severely ill before traveling to a clinic [70]. As a result, patients are unlikely to make it to a hospital, receive an official diagnosis, and begin effective ribavirin treatment within the “therapeutic time window”. This leads to patients requiring more intensive medical intervention and accumulating higher associated medical bills. A 2016 report indicated that, on average, unsubsidized treatment for LF can cost a patient USD 460.35 and an average of USD 194.40 when subsidized [70]. Even when subsidized, this cost is disproportionately higher than the typical income in Nigeria, with the average subsidized cost being 289.34% of the NGN 30,000 monthly minimum wage in 2022 [71]. In an effort to reduce the burden of LF management, the government has recently been providing ribavirin free to patients at designated treatment centers [72]. Nevertheless, supportive management of LF cases, including blood transfusion, dialysis, and other measures, may be considerably more expensive than the cost of ribavirin. In comparison to other diseases endemic to the area, the out-of-pocket burden is markedly higher for LF patients [70]. For example, a 2015 study indicated that patients seeking treatment for malaria pay on average USD 22.90 out of pocket [73]. Another 2013 study indicated that total treatment costs for diseases including malaria, typhoid, diarrhea, and other diseases ranges from USD 7.90 per month to USD 25.60 per month [74]. Therefore, despite there being an effective treatment for LF, case fatality rates remain high due to poor access to healthcare facilities and the overall cost of LF treatment resulting in prolonged time to hospitalization and gaps in disease management.

Despite clinical reports touting the effectiveness of ribavirin, case fatality rates observed during LASV outbreaks remain high in part due to the high cost of treatment. The low incidence but high case fatality rates of LASV infection suggest that a greater benefit to public health could be observed by increasing accessibility to therapeutics. Therefore, significant effort should also be placed on revamping the healthcare system to make ribavirin readily available and affordable for rapid deployment in outbreak situations. This would allow early treatment of outpatients to become a possibility, potentially reducing hospitalization and death. We believe that focus should be placed on either remedying the problems associated with ribavirin and/or the development of more LASV antivirals to help control viremia, while carefully testing vaccine candidates in clinical trials.

## 4. Sensorineural Hearing Loss

There are three types of hearing loss: sensorineural, conductive, and mixed hearing loss. Sensorineural hearing loss (SNHL) occurs upon damage to the auditory nerve or within the inner ear, whereas conductive hearing loss occurs when sound is not transmitted effectively through the middle or outer ear, and mixed hearing loss occurs when both types of hearing loss are present simultaneously [75]. Causative factors for SNHL include aging, genetics, exposure to ototoxic drugs, injury, and infection [75]. Sudden SNHL is a diagnosis given to a condition with a rapid decrease in hearing of 30 decibels or more affecting at least three consecutive frequencies within a 72 h window. Sudden SNHL occurs without an identifiable cause in 90% of cases [76]. However, among known causes, it is commonly related to infection and is rarely able to be reversed [77,78,79]. While steroid treatment may allow for some improvement of SNHL, neither surgical nor medical interventions are typically helpful [75,78,80].

Sudden-onset SNHL is the most common sequela of LF, occurring in approximately one-third of patients, with two-thirds of these cases being permanent [26,81,82]. This LF-associated SNHL may occur as unilateral or bilateral, mild to profound in degree, develop during either the late stage of disease or early convalescence, and it can occur in both clinical and subclinical cases [81]. As over 80% of LASV infections are either subclinical or undiagnosed, thousands of individuals likely develop this hearing loss without knowing the cause [14,16,26,83]. Treatment with ribavirin neither slows nor reverses hearing loss progression [81]. No other infectious disease has such a staggering impact on hearing ability. While other viruses feature SNHL as a common sequela, including measles, cytomegalovirus (CMV), and human immunodeficiency virus (HIV), the incidence is significantly lower [84,85,86,87].

LF-associated SNHL generates a huge socioeconomic burden throughout western Africa [26]. Moderate or greater degrees of SNHL are not easily hidden, and survivors often face stigmatization and isolation, leading to increased rates of depression and unemployment [83]. This generates a large financial burden on countries, costing Nigeria alone upwards of USD 43 million each year [26]. This burden disproportionately affects children, as they are hindered both by the social stigmatization and the lack of rehabilitation services, impacting speech development and educational progress [83,88].

Despite the prevalence of this sequela, the exact mechanism behind LF-associated SNHL remains unknown. Prior research, conducted by our lab and others using a reliable and reproducible murine model of LF-associated SNHL, implicated an immune-mediated mechanism inducing damage within the inner ear [27,89]. This hypothesis was further confirmed using a nonhuman primate model [90]. More recently, studies using the murine model have demonstrated LF-associated SNHL occurs in a CD4 T cell-dependent manner, with depletion of CD4 T cells preventing hearing loss [27]. Although the exact mechanism of this immune-mediated hearing loss has yet to be elucidated, current hypotheses speculate that during the acute phase of infection, lymphopenia and viral antagonism of the interferon response allow for prolonged LASV infection, resulting in viral dissemination and incomplete clearance from the inner ear [20,27]. Recovery of immune cell populations in the convalescent phase then leads to SNHL development through either recognition of persistently infected cells or autoimmunity induced by molecular mimicry [27]. A major implication of this hypothesis is that administration of LASV antigens mimicking self-epitopes could lead to the development of an autoimmune response causing SNHL as a result of vaccination. Developing a vaccine that may induce SNHL could have devastating impacts, not only on patient wellbeing but on the public perception of healthcare as a whole. The greatest public health benefit would come from a vaccine that prevents both acute disease associated with significant morbidity as well as the development of hearing loss.

This autoimmune-associated mechanism of SNHL is not unique to infectious diseases. Many autoimmune disorders themselves include a hearing loss component [91,92,93,94]. However, there are no animal models of infectious diseases that consistently induce SNHL apart from the LF murine model [27,89]. Further investigation into the immune-mediated mechanism driving LF-associated hearing loss using this model will be essential to fully understand the risks associated with LASV vaccination. In turn, this will contribute significantly to the understanding and development of therapeutics for numerous infectious diseases and autoimmune disorders.

These recent findings and current hypotheses raise significant concerns regarding vaccine development. Vaccinations that do not prevent infection and viral dissemination or that introduce an antigen that induces the development of an autoimmune response may enhance the risk of developing SNHL [27]. Therefore, understanding the mechanism leading to LF-associated SNHL is crucial prior to vaccine administration in humans. Considering other endemic diseases in the region, LF poses a lesser risk to public health in western Africa. Investments in infrastructure such as access to clean water or sustainable electricity supply would have a strong health impact in West Africa.

## 5. Current Vaccine Candidates

While there are currently no FDA-approved vaccines against LASV, at the time of writing, there are three vaccine candidates in phase I clinical trials. These include a recombinant measles virus (MeV) expressing the LASV glycoprotein (GPC) and nucleoprotein (NP), a recombinant vesicular stomatitis virus (VSV) expressing the LASV GPC, and a DNA vaccine encoding the LASV GPC named INO-4500 [95,96,97]. While the various vaccine platforms offer their own risks and benefits, an ideal LASV vaccine candidate would offer life-long efficacy after a single dosage and would cross-protect against the seven currently identified lineages of LASV.

The live attenuated measles vaccine platform has been in use since the 1960s and is well tolerated in recipients with easy production, high efficacy, and high immunogenicity [98,99]. MeV-LASV, a live attenuated vaccine including the Lineage IV NP of LASV, has proven to have nearly sterilizing immunity in a cynomolgus macaque model after a single dose [99]. The efficacy of this vaccine was maintained for both Lineages II and VII as well [100]. The long-term efficacy and single dose of this vaccine make it an ideal candidate for use in endemic areas, mitigating the difficulties with patient follow-up and vaccine administration. MeV-LASV concluded phase I clinical trials in January 2021, although the results are not yet published [95].

The recombinant VSV (rVSV)-vectored vaccine platform has previously been used for other infectious diseases and is currently the sole FDA-approved vaccine against Ebola virus (EBOV) [101]. The rVSV platform has been well characterized and induces high immunogenicity through both a cellular and humoral response; pre-existing immunity against VSV has been shown to not be an issue [102,103,104,105,106,107,108,109,110,111]. Pre-existing immunity to the VSV vector platform is rare due to very low levels of VSV seropositivity in the human population [112]. For vaccination strategies employing a prime-boost model, pre-existing immunity can be circumnavigated by utilizing a different VSV serotype as the vector [113]. However, a recent study showed that vaccination with rVSV expressing Marburg glycoprotein did not prevent efficacy of rVSV expressing EBOV glycoprotein, suggesting that the presence of pre-existing VSV immunity does not affect vaccines employing this vector [114]. The rVSVΔG platform is further attenuated by removing the glycoprotein (G) of VSV, which is responsible for the neurologic spread of VSV; this also allows for the insertion of the viral glycoprotein of interest [115]. rVSVΔG-LASV has been shown to be safe and efficacious in mice, guinea pigs, and cynomolgus macaques over multiple viral lineages [116,117,118,119]. However, a low-level viremia has been noted in the macaque model [120]. Although safe, a series of mild adverse events such as myalgia, headache, fatigue, and injection site pain may be associated with the VSV-based vaccines, as seen with the recently approved rVSV-ZEBOV vaccine [121]. This vaccine is currently in the recruiting phase of clinical trials with an estimated study completion date of February 2024 [96].

INO-4500 is a DNA vaccine that was developed in response to the low-level viremia seen with rVSVΔG-LASV in the cynomolgus macaque model [120]. The plasmid vector encodes the GPC of LASV Josiah and has been shown to be highly efficacious in both guinea pigs and cynomolgus macaques [120,122,123,124]. This vaccine has completed phase I clinical trials, but the results have yet to be made available [97]. Despite promising efficacy data, there are several considerations that must be made about the realistic application of DNA vaccines, including limited access to the equipment required for vaccination in LASV-endemic areas as well as the general difficulty of follow-up vaccination. However, DNA vaccines are also known to be markedly thermostable, which makes them ideal for western Africa, where maintaining a cold chain may be difficult.

ML29 is a live attenuated vaccine candidate against LASV that has yet to reach a clinical trial phase [125]. ML29 is a reassortant virus, composed of the small (S) segment of the Josiah strain of LASV and the large (L) segment of the AN20410 strain of a closely related nonpathogenic Old-World arenavirus, Mopeia virus [125]. ML29 has demonstrated immunogenicity in murine models, with the robust T cell response hypothesized to be the correlate of protection [125,126]. However, use in a signal transducer and activator of transcription-1 knockout (STAT1^−/−^) model demonstrates high pathogenicity and lethality [126,127]. Inoculated strain 13 guinea pigs have also indicated the protective nature of ML29 against LASV infection [125,128,129]. Rhesus macaque and common marmoset models have been used to demonstrate vaccine safety and efficacy, with inoculated primates developing no clinical, serological, or histological signs of disease and high immunogenicity [125,130]. Additionally, simian immunodeficiency virus (SIV)-infected macaques develop no clinical signs upon infection with ML29, indicating its potential as a safe candidate in populations with high rates of HIV [131].

Of these listed vaccine candidates, ML29 alone has been tested for its safety in regard to the development of SNHL in the mouse model [127]. Unfortunately, the pathogenicity of recombinant ML29 in STAT1^−/−^ mice does not allow for studying sequelae in survivors, although a series of T cell depletions indicates that ML29-associated hearing loss occurs in a T cell-independent mechanism [127]. While this mechanism leading to hearing loss is different than that of LASV, it raises further concerns about vaccine safety and emphasizes the critical need to thoroughly investigate the potential of each vaccine candidate to induce SNHL before introducing them into human populations.

Another obstacle facing LASV vaccine development is the variation between viral lineages. LASV is phylogenetically divided into seven lineages, with each lineage being predominant in certain countries or regions in western Africa [89,132,133,134,135]. These lineages are extremely diverse, and vaccine candidates must be tested for efficacy against each. This strain diversity increases the likelihood that developing a universal vaccine against LASV is unlikely unless multiple antigens are used. Additionally, this elevates the risk of not understanding full mechanisms of protection in humans required for vaccine design. The existence of multiple lineages and the associated challenges to vaccine design provide additional reasons to focus on developing rapid diagnostics and early treatments that are accessible and affordable.

When developing vaccines, the realities of the environment in which they would be used must also be taken into consideration. The unstable electricity throughout western Africa renders any vaccine platform that requires refrigeration or freezing for storage ineffective. Moreover, maintaining a cold chain throughout the shipment of these vaccines is unlikely. Using DNA-vectored vaccine platforms that require electroporation devices for delivery is also not necessarily feasible, as the equipment may not be attainable for most health clinics. Factoring in these hurdles is crucial when contemplating a vaccination drive as opposed to wasting time and resources on generating a platform that cannot feasibly be deployed within the target regions.

## 6. Conclusions

The widely accepted incidence of LASV infection in western Africa is between 100,000 and 300,000 cases annually, based on serial publication of extrapolated data. In this review, we summarize the reported case numbers from Nigeria, a LASV hot spot, that suggest these published statistics are likely overestimated. In accordance with recent data, between 1969 and 2017, the highest annual number of reported LF cases in Nigeria was still only 1022 cases during the 2016–2017 outbreak [33]. More recently, in 2020, this number increased to 1189 [11], still vastly below the American CDC estimates of hundreds of thousands. While the incidence of LASV infection may be lower than currently thought, reports from Nigeria indicate the case fatality rate associated with LF is significantly higher than the widely published 1%. Between 1969 and 2021, the overall case fatality rate associated with LASV infection in Nigeria was 33.8% [32]. This decreased incidence but heightened risk of mortality upon infection suggests that therapeutic measures, rather than preventative measures such as vaccines, could have the greatest impact on increasing LF survival.

Currently, the only therapeutic used in the treatment of LF is off-label administration of ribavirin, which has been shown to be highly effective in both animal and clinical studies in reducing mortality [23,28,48,49,50,52,53,54,55,58,59,60]. Treatment of LF patients within the first six days post onset of symptoms significantly reduces viremia, which has been correlated with patient survival [23,28,48,49,50,52,53,54,55,58,59,60]. Despite the effectiveness of this therapeutic, case fatality rates remain high due to the inaccessibility of ribavirin and late presentation of patients to clinics. Even with government subsidization, the average cost of LF treatment is USD 194.40, 289.34% of the Nigerian monthly minimum wage [70,71]. As a result, LF patients avoid seeking treatment until their symptoms are severe, at which point they are likely outside the effective time window for ribavirin treatment. Diverting research and development efforts from preventative vaccines to therapeutic antivirals as well as focusing on ribavirin efficacy, availability, and affordability would lead to a greater public health benefit.

The reality of lower incidence rates and the presence of an effective therapeutic allows for careful and well-planned vaccine development to target acute disease as well as LASV-induced hearing loss. However, it is also very important to minimize any potential adverse events, including “vaccine-induced hearing loss”. Our major concern made evident throughout this review is the risk of vaccination inducing similar immune-mediated pathology as that seen in infection. This could put vaccine recipients at a high risk of SNHL. Although this is currently only our speculation, recent research strongly supports the finding that LASV-associated hearing loss is induced by the T cell response against infection; these T cells induce inner ear damage in the mouse model as a result of either viral persistence or autoimmunity due to molecular mimicry [27,89]. In either case, this has serious implications for vaccine design. Moreover, if the initial destruction of the inner ear cells seen upon LASV infection drives the induction of a certain immune memory, then in any later case of severe ear infection or usage of ototoxic drugs that cause inner ear damage, the resultant inner ear inflammation and autoimmune antigens could enhance the inner ear damage resulting in hearing loss. Due to the fact that the mechanism driving LASV-associated hearing loss is largely unknown, it is critical that vaccine development and administration be conducted with an abundance of caution.

Our objective in this article is to describe the current situation concerning LF in Nigeria and West Africa and to emphasize the need for developing a safe and effective vaccine that would protect against acute diseases as well as against the development of hearing loss. As LASV does not present a severe public health burden in comparison to other infectious diseases endemic to Africa, investment into rapid diagnostics and antiviral therapeutics while conducting further research on LF and its associated hearing loss should also be prioritized. Any vaccine candidates must be thoroughly safety-tested in animal models prior to a roll out to ensure there is no risk of inducing hearing loss or other sequelae in recipients. Moreover, resources must be invested into fully understanding the mechanisms driving hearing loss to ensure novel and safe vaccines can be generated. Alternatively, investments into the development of antivirals and other more pressing issues such as access to clean water and sustainable electricity supply would strongly benefit public health in West Africa.

## Figures and Tables

**Table 1:** Comparing yearly case numbers and fatalities due to diseases of public health importance in Nigeria. Numbers represent approximated annual cases and deaths based on currently available data cited in the reference column.

Public Health Impact of Diseases Endemic to Nigeria			
Disease	Cases per Year	Deaths per Year	Reference
Lassa Fever	500 (suspected)	25	[31]
	75 (confirmed)		
Malaria	100,000,000	300,000	[38]
Yellow Fever	4000 (suspected)	112	[43]
	140 (confirmed)		
Cholera	3000	781	[46]
Dengue	Published case numbers have not been reported but high seropositivity (10–67%) has been observed in Nigerian populations		[40,41,42]

## Data Availability

No new data were created or analyzed in this study. Data sharing is not applicable to this article.
